# Acupuncture Mechanism and Redox Equilibrium

**DOI:** 10.1155/2014/483294

**Published:** 2014-07-07

**Authors:** Xiang-Hong Zeng, Qian-Qian Li, Qian Xu, Fang Li, Cun-Zhi Liu

**Affiliations:** ^1^Acupuncture and Moxibustion Department, Beijing Hospital of Traditional Chinese Medicine Affiliated to Capital Medical University, 23 Meishuguanhou Street, Dongcheng District, Beijing 100010, China; ^2^Acupuncture and Moxibustion College, Tianjin University of Traditional Chinese Medicine, No. 312, Anshan West Road, Nankai District, Tianjin 300193, China

## Abstract

Oxidative stress participates in the pathological process of various diseases. Acupuncture is a component of the health care system in China that can be traced back for at least 3000 years. Recently, increased evidences indicate that acupuncture stimulation could reduce oxidative damage in organisms under pathological state, but the exact mechanism remains unclear. This review focuses on the emerging links between acupuncture and redox modulation in various disorders, such as vascular dementia, Parkinson's disease, and hypertension, ranging from redox system, antioxidant system, anti-inflammatory system, and nervous system to signaling pathway. Although the molecular and cellular pathways studies of acupuncture effect on oxidative stress are preliminary, they represent an important step forward in the research of acupuncture antioxidative effect.

## 1. Introduction

Oxidative stress is defined as the imbalance between the production of reactive oxygen and nitrogen species (ROS/RNS) and the endogenous antioxidant system, causing a cascade of chain reactions resulting in cellular damage and disease. Under physiological conditions, several related oxidative pathways contribute to ROS/RNS productions, while several intra- and extracellular antioxidant enzymatic systems account for ROS/RNS elimination [1]. Therefore, oxidative stress is a critical feature in the pathological process of various diseases. ROS/RNS is responsible for direct damage to cellular structures, while it also triggers a shift in the redox state of the biological compartment towards one that is more oxidizing [2, 3].

As one of the traditional oriental medicines, acupuncture has been widely used for more than 3000 years as a treatment for many diseases [4]. Recently, a large body of evidences demonstrated that acupuncture has antioxidative effect in various diseases [5–7], but the exact mechanism remains unclear. A literature review was conducted using Pubmed. Keywords were “acupuncture,” “electric acupuncture (EA),” “acupoint,” or “moxibustion” in combination with “antioxidative,” “oxidative stress,” “reactive oxygen species (ROS),” “reactive nitrogen species (RNS),” “redox,” or “free radicals.” The records were collected from December 2008 to present in each database. A total of 117 publications were identified as a result of the search which was related to acupuncture study and redox modulation. Eighty-four articles met the criteria; 79 of the articles in English and 5 articles in Chinese. In this review, the underlying mechanism of acupuncture-induced antioxidative effect is discussed based on the studies that have been published in the last 5 years. We will, in particular, focus on the antioxidative effect of acupuncture on (1) vascular dementia (VD); (2) Alzheimer's disease (AD); (3) Parkinson's disease (PD); and (4) hypertension.

## 2. Vascular Dementia 

Changes in free radical generation and consequent oxidative stress may have a role in the pathogenesis of ischemic lesions. It has now been well established that generation and accumulation of ROS are detrimental to cells* in vitro* and* in vivo* [8] and promote cell death [9]. VD-induced damage of neural tissues has been proved to produce excessive ROS [10]. It has been reported that acupuncture could improve memory impairment in VD patient [11]. The cognitive enhancing effect of acupuncture is likely to be at least partially attributable to decreased oxidative stress [12]. Shi et al. [13] found that oxidative stress marker 8-hydroxydeoxyguanosine (8-OHdG) increased significantly in the urine of VD patients. Meanwhile, the content of 8-OHdG could be decreased and cognitive function and quality of life were improved after acupuncture treatment [14]. Experimental studies reported that EA could effectively attenuate lipid peroxidation and malondialdehyde (MDA) content through increasing the antioxidant enzyme activities, such as superoxide dismutase (SOD) and glutathione peroxidases (GSH-Px), in hippocampal CA1 of VD rats [15, 16]. Consisting in these results, Liu et al. [7, 17] found that acupuncture could improve memory impairment through increasing antioxidant system ability, especially the expressions of CuZnSOD, and redox effector factor (Ref-1) in the hippocampus of VD rats. Furthermore, Zhang et al. [18] found that acupuncture's improvement of cognitive abilities was contributed to elevate MnSOD activities and the ratio of reduced glutathione (GSH) and oxidized glutathione (GSSG) in mitochondria in MID rats. These observations indicate that acupuncture-induced antioxidative effect may be related to GSH system and antioxidant enzyme in hippocampus which is crucial to learning and memory formation in vascular dementia patients and models.

Besides GSH system, a huge production of ROS during ischemia reperfusion alters a properly balanced thiol-redox environment, resulting in the oxidation of protein thiols of some enzymes and a loss of their normal biological activities [19, 20]. Thioredoxin (Trx) system, consisting of thioredoxin reductase (TR), Trx, and Nicotinamide Adenine Dinucleotide Phosphate (NADPH), could prevent susceptible proteins from this oxidative modification [21, 22]. Siu et al. [23] suggested that electroacupuncture (EA) treatment at* Zusanli* (ST36) could increase Trx expression in ischemic-reperfused brain tissues, which in turn increase the activity of antioxidase, shifting the intracellular more oxidative state to redox balance, and subsequently suppress ROS production. However, there are limited researches about the acupuncture effect on TR and NADPH, and further studies need to be performed.

During the subacute phase of ischemic brain injury (1–7 d after the onset of ischemia), astrocytes become activated and accumulate in the peri-infarct area, leading to glial scar formation. Complex neuron-glial interactions at high concentration could upregulate inducible nitric oxide synthase (iNOS) expression and nitric oxide (NO) production in glial cells, causing NO diffusion and neurotoxicity which exacerbate delayed infarct expansion and play a key pathological role in ischemic injury [24]. Increasing evidences have suggested that the neurotoxic effects of iNOS-derived NO could be attributed to its combination with the superoxide anion, leading to the formation of peroxynitrite, a strong oxidative/nitrative molecule that aggravates cerebral ischemia/reperfusion (I/R) injury [25]. EA could effectively downregulate astrocytic S100B expression to provide neuroprotection against delayed infarct expansion by modulating p38 MAP kinase-mediated NF-*κ*B expression. These effects could subsequently reduce oxidative/nitrative stress and inhibit the TNF-*α*/TRADD/FADD/cleaved caspase-8/cleaved caspase-3 apoptotic pathway in the ischemic cortical penumbra 7 d after reperfusion [26]. This suggests that EA could reduce oxidative/nitrative stress and NF-*κ*B-mediated inflammation during the later stages of cerebral I/R injury.

## 3. Alzheimer's Disease

AD is the most common form of dementia, which is characterized by the deposition of the amyloid *β* (A*β*) peptide and microtubule-associated protein tau in the brain [27, 28]. It has been proved that A*β* has capacity to interact with transition metals generating redox active ions, which precipitate in lipid peroxidation and cellular oxidative stress [29]. In other words, A*β* promotes cellular oxyradicals accumulation in neurons and glial cells in vulnerable regions of AD brain. Such oxidative stress may lead to many of the metabolic and neurodegenerative alterations observed in this disease. A variety of markers of oxidative stress are increased in postmortem brain tissues of AD patients, with a clear relationship with A*β* deposition and neurofibrillary degeneration [30]. It has been reported that the activity and/or protein levels of several antioxidant enzymes were altered in AD brain regions, consistent with ongoing oxidative stress [31].

Acupuncture has been reported to improve intelligence and ameliorate depression and anxiety in AD patients [32, 33]. This effect may be through decreasing the A*β* proteins level and increasing antioxidant system SOD and GSH-Px activities in the hippocampus of AD rats [34, 35]. APP transgenic mice study showed that EA stimulation at 2 Hz/100 Hz could significantly improve learning-memory capacity by reducing the expression of A*β* precursor protein and A*β* protein in the cerebral cortex and minimizing neuronal mitochondrial damages in hippocampal CA1 region [36]. Besides, EA at specific acupoints could improve the cognitive function of senescence-accelerated mouse (SAMP10) through reversing age-related protein and gene expression profiles, such as Hsp84, Hsp86, and YB-1, which are closely involved in oxidative stress-induced damage in the hippocampus [37]. Another research suggested that Choline acetyltransferase (ChAT) and acetylcholinesterase (AChE) activities of hippocampal tissues were decreased in AD rat and acupuncture stimulation could reverse the decrease in both ChAT and AChE [38]. IPF2a, a sensitive and specific marker of lipid peroxidation, has been considered to be correlated with the cognitive functional impairment in AD patients [39]. The clinical research has reported that acupuncture stimulation could decrease iPF2a level in the cerebrospinal fluid, blood, and urine in AD patients [40].

Microglia is functionally polarized into different phenotypic activation states, referred as classical and alternative. The balance of the two phenotypes may be critical to ensure proper brain homeostasis and may be altered in brain pathological states of Alzheimer's disease. Inhibition of NADPH oxidase or gene deletion of its functional p47phox subunit switched microglial activation from a classical to an alternative state in response to an inflammatory challenge, representing a promising neuroprotective approach to reduce oxidative stress and modulate microglial phenotype towards an alternative state [41]. Previous studies suggested that acupuncture could regulate microglial activation and attenuate oxidative stress in Limb ischemia reperfusion [42] and spinal cord injury [43], but that is not reported in Alzheimer's disease, which is worthy of further research. These observations suggest that the effects of acupuncture on learning-memory capacity in AD rats/patients may be related to neuronal mitochondrial integration, A*β* plaques, and acetylcholine neurotransmission.

## 4. Parkinson's Disease (PD)

Oxidative stress is thought to be one of the primary mechanisms behind the onset and progression of the neurodegeneration in PD [44], as highly neurotoxic free radicals are generated through the metabolism of dopamine and its own autooxidation [45]. Increased oxidative stress and mitochondrial dysfunction have been shown in PD patients. In particular, patients have disrupted iron (Fe) metabolism, as well as altered mitochondrial energetics, with a decrease in mitochondrial complex I levels and overall oxidative phosphorylation in the substantia nigra (SN) [46]. Moreover, depletion of the antioxidant glutathione (GSH) is also a prominent molecular consequence in PD, with several recent studies focusing on the potential therapeutic benefits of GSH administration [47].

Recent studies suggested acupuncture could attenuate oxidative stress and inhibit cell death in SN dopaminergic neurons [48]. The antioxidative effect of acupuncture is mediated by the activity of antioxidant system, inhibiting the production of H_2_O_2_ and MDA in 6-OHDA-lesioned rats or 1-methyl-4-phenyl-1,2,3,6-tetrahydropyridine (MPTP) mouse [49, 50]. Further study displayed that acupuncture could counteract MPTP-induced increase of oxidative stress-related proteins, such as cytosolic malate dehydrogenase, hydroxyacylglutathione hydrolase, and cytochrome c oxidase subunit Vb [51].

The iron redox system in SN may contribute to the vulnerability of dopaminergic neurons [52, 53]. Some reports have also shown that Fe3+ accumulated in the SN region of PD patients [54], MPTP-treated mice [55], and 6-OHDA-treated rats [56]. Acupuncture stimulation at* Taichong* (LR3) and* Yanglingquan* (GB34) acupoints for 15 days could attenuate MPTP-elicited dopaminergic neuronal degeneration through lowering levels of Fe3+ and ferritin-heavy chain, suggesting that acupuncture treatment could attenuate iron-related oxidative damage to dopaminergic neurons [57].

Phosphatidylinositol-3-kinase/protein kinase B (PI3K/Akt) signaling pathway is necessary for neuroprotection against MPTP-induced oxidative damage [58]. Acupuncture could restore the MPTP-induced impairment of Akt activation in SN dopaminergic neurons [59]. Furthermore, acupuncture-induced dopaminergic neuron protection and motor function improvement were significantly blocked by administration of LY294002, a specific inhibitor of PI3K/Akt signaling pathway [60]. These studies provide evidences that PI3 K/Akt signaling pathway may play an important role in the mechanism underlying acupuncture-induced neuroprotection in PD mouse.

Acupuncture has been proved to significantly reduce rotational motor deficit in Parkinson model through increasing expression of brain-derived neurotrophic factor (BDNF) receptor [61] and reducing TNF/IL-1 beta mRNAs and macrophages in the ventral midbrains [62]. These results concluded that neuroprotection by acupuncture treatment comes from the collaboration of its anti-inflammatory actions. Reactive microglia was present in the brains of Parkinson's disease [63]. It will be of interest to see whether acupuncture treatment prevents microglial activation for neuronal degeneration.

## 5. Hypertension

Oxidative stress in the central nervous system has an important role in the neural mechanisms of hypertension [64]. Brain stem ROS acts on the rostral ventrolateral medulla (RVLM) or nucleus tractus solitarii (NTS), augmenting central sympathetic outflow and suppressing baroreflex regulation of blood pressure [65]. Significance of the NADPH oxidase-derived ROS in pathogenesis of hypertension was comprehensively discussed in several recent reviews [66, 67]. In hypertension, neurohumoral stimuli such as Ang II, NE, and ET-1 activate receptors located on cell membrane, namely, AT1, *α*-AR, and ET receptors. The function of these receptors is coupled to G proteins, which activate the source of ROS, NADPH oxidase. The activated NADPH oxidase will produce ROS (e.g., O_2_^−^), and these, in turn, activate cell phosphorylation pathways: the mitogen-activated protein kinases (MAPKs), tyrosine kinases, and phosphoinositol-3-kinase/Akt kinase (PI3K/Akt). The activated phosphorylation pathways activate transcription factors, such as activated protein-1 (AP-1), p53, nuclear factor kappa B (NF-*κ*B), and nuclear E2-related factor 2 (Nrf2), which stimulate transcription of genes after moving into nucleus. Proteins encoded by these target genes in turn mediate cellular consequences leading to changes in the phenotypes, such as hypertrophy, inflammation, necrosis, and apoptosis of cells and, on the other hand, stimulate the production of antioxidants involved in antioxidant defense [68].

Emerging evidences indicate that acupuncture could regulate blood pressure in hypertensive patients [69, 70]. The antihypertensive effect of acupuncture is related to the expression of different NOS, especially eNOS and iNOS in the RVLM of stress-induced hypertensive rats. Acupuncture could decrease blood pressure by increasing antioxidant enzymes, such as glutamate dehydrogenase 1, aldehyde dehydrogenase 2, glutathione S-transferase M5, and SOD in the medulla of the SHRs [71]. The antihypertensive effects of EA might be associated with the attenuation of apelin expression in the RVLM, exerting its anti-inflammatory effects, and then downregulated the apelin-induced oxidative stress [72]. Besides, an animal model of renal failure- (RF-) induced hypertension study showed that the antihypertensive mechanism of EA may be related to the effects of oxidative stress on insulin-like growth factor-I (IGF-I), inducible nitric oxide synthase, heme oxygenase, and thiobarbituric acid-reactive substance expression [73]. These views suggest that antihypertensive effects of acupuncture may be mediated by antioxidant enzymes and anti-inflammatory effects, which could modulate the renal sympathetic nerve activity and nitric oxide levels, leading to decreased blood pressure.

## 6. Other Diseases 

In fact, the antioxidative effect of acupuncture has been verified in other diseases. Hyperglycemia and oxidative damage were relieved after preventive acupuncture by reducing LPO level and enhancing SOD activity in the serum and the pancreas of streptozotocin-induced hyperglycemia rats [74]. More studies confirmed that EA exerts its antioxidative effect through inducing nNOS and iNOS expressions which are involved in NO signal transduction and increasing total NO concentration in hypercholesterolemia rats [75], acetylsalicylic acid-induced acute gastritis rats [76], and LPS-induced kidney injury rats [77].

Through detecting the activity of antioxidant system, many researches suggest that acupuncture could reduce oxidative damage in different organs and tissues, such as plasma and ovary [78], liver and kidneys [79], hypothalamus [80], and random skin flaps [81] in estradiol-induced inflammation and oxidative stress rats. The analgesic effect of acupuncture was mediated by inhibiting the production of superoxide anion (O_2_^−^) and ROS-induced p38MAPK and extracellular signal-regulated kinase (ERK) activation in microglia of spinal cord injury rats [82]. Furthermore, Moore and Roberts II [83] demonstrated that the removal of intracellular superoxide at acupoints may be an important process in reduced simple adiposity.

## 7. Conclusions 

In conclusion, oxidative stress is an essential pathophysiological change of various diseases, but it serves as a potential treatment target. The above accumulating evidences demonstrates that acupuncture plays an antioxidant effect on these diseases. Through redox system, antioxidant system, anti-inflammatory system, nervous system, and signaling pathway, acupuncture could make the oxidative damage and antioxidant defense remain relatively constant redox state. However, the recent acupuncture researches about oxidative stress are sporadic and preliminary, and further thorough studies on possible antioxidative actions of acupuncture are highly recommended, especially the influence of acupuncture on signaling pathways. The corresponding research on new therapeutic targets may be helpful to our understanding about the mechanism of acupuncture.
